# Editorial: Recent advances in causes, diagnosis, and therapeutics for congenital heart defects

**DOI:** 10.3389/fgene.2025.1564492

**Published:** 2025-02-18

**Authors:** Xinxiu Xu, Cecilia W. Lo, Lisa J. Martin, Lu Han, Dongzhu Xu

**Affiliations:** ^1^ Department of Pathology, Microbiology and Immunology, Vanderbilt University Medical Center, Nashville, TN, United States; ^2^ Department of Pediatrics and Department of Developmental Biology, University of Pittsburgh, Pittsburgh, PA, United States; ^3^ Division of Human Genetics, Cincinnati Children’s Hospital Medical Center, Cincinnati, OH, United States; ^4^ Department of Pediatrics, College of Medicine, University of Cincinnati, Cincinnati, OH, United States; ^5^ Department of Pediatrics, Medical College of Wisconsin, Milwaukee, WI, United States; ^6^ Division of Pediatric Cardiology, Herma Heart Institute, Children’s Hospital of Wisconsin, Milwaukee, WI, United States; ^7^ Department of Cardiology, Institute of Medicine, University of Tsukuba, Tsukuba, Japan

**Keywords:** heart development, congenital heart disease, genetic causes, diagnosis, evidence-based therapies

## Introduction

Congenital heart disease (CHD) is one of the most prevalent major birth defects, yet its causes remain largely unknown. Both genetic and environmental factors play a role. Animal and human induced pluripotent stem cell models have shown how these factors disrupt heart development (Liu et al., 2017; Xu et al., 2022), but the precise mechanisms in humans remain unclear.

Advanced genetic and genomic approaches have significantly improved CHD diagnosis and therapies, especially through prenatal genetic testing, enabling earlier and more accurate diagnosis and screening. As survival rates into adulthood improve, new research directions have emerged, including exploring the genetic basis of surgical outcomes and developing therapies to enhance the quality of life for CHD patients. The growing population of adults with CHD, who lacked access to modern genetic technologies during childhood, underscores the need for ongoing research and tailored medical care (Bhatt et al., 2015).

This Research Topic encompasses a total of 14 articles, including basic research studies, clinical case reports, and a mini review. The novel findings focus on both pediatric and adult CHD (ACHD), covering recent advances in the causes, diagnosis, and therapeutics of CHD. These studies collectively demonstrate that integrating genetic data with clinical assessments provides crucial insights for developing precise diagnostic and therapeutic strategies.

## New causes and biomarkers in heart development abnormalities

The identification of novel genetic markers and pathways is a pivotal advancement in CHD research. Several studies in this Research Topic revealed novel biomarkers and genetic factors in cardiac developmental abnormalities. Hu et al. have reported that the structural protein Sorbs2 promotes the development of the second heart field, with its deficiency resulting in atrial septal defects. Their continued research found increased cardiac macrophages in Sorbs2-deficient hearts, revealing a potential role for macrophages in responding to embryonic myocardial abnormalities. A significant breakthrough came from Xu et al., who identified Keratin 19 (Krt19) as a novel epicardial gene through cardiac single-cell mRNA sequencing analysis. This discovery provides valuable insights into epicardial contribution to embryonic and neonatal heart development, addressing a long-standing challenge in the field. Additionally, Li et al. identified novel markers associated with increased risk and pathogenesis of immune checkpoint inhibitor-associated myocarditis through serum autoantibody profiles, expanding our understanding of cardiac complications in immunotherapy.

## Genetic diagnosis in human congenital heart disease

The application of advanced genetic and genomic technologies, such as microarray testing and next-generation sequencing (NGS), has revolutionized the diagnostic landscape of CHD. Mascho et al. found an association between left outflow tract obstruction and 5p deletion, with high mortality in the presence of additional copy number variants. Similarly, Yu et al. used microarray analysis to identify both *de novo* and inherited micro-CNVs at 16p13.11 in 21 Chinese patients with defective cardiac left-right patterning. Significant progress in understanding specific CHD subtypes emerged from several studies. Li et al. conducted whole exome analysis of 25 Patent foramen ovale (PFO) patients, identifying potential mutant genes like *LDLR, SDHC*, and *NKX2-5*, enhancing our understanding of PFO’s genetic basis. Chen et al. explored the impact of a *KCNH2* missense variant on Long QT syndrome, revealing incomplete penetrance influenced by sex, potentially shedding light on the distinct penetrance behaviors and patterns of the *KCNH2* gene. In another study, Wang et al. studied complex arrhythmias in children with *RYR2* gene sequence variation, providing evidence for early detection and treatment. Additionally, Shen et al. reported a pathogenic MEIS2 sequence variation in a patient with multiple conditions, expanding the CHD symptom spectrum associated with *MEIS2* sequence variations.

## Potential therapeutics and new challenges

New research and advances in genetic diagnostics have led to evidence-based therapies. This Research Topic highlighted several promising therapeutic developments and identified critical challenges in CHD management. Dyrka et al. showed the efficacy and safety of long-term recombinant growth hormone treatment on aortic dimensions in a girl with Loeys-Dietz Syndrome. Hiraya et al. identified implications for diagnostic strategies and therapeutic approaches by performing genetic testing and human leukocyte antigen analysis in patients with hypertrophic cardiomyopathy and connective tissue diseases.

Improved management of CHD has increased the life expectancy of adults with CHD. Many adults missed out on genetic evaluations during childhood due to the lack of modern genetic technologies. Oehlman et al. highlighted the need for guidelines to enhance access to genetic services and improve medical management by studying how ACHD cardiologists offer genetic services. Edwards et al. revealed significant gaps in genetics-related care, especially for those ACHD patients with congenital and neurodevelopmental comorbidities, through a retrospective chart review.

The identification of neurological deficits in adult CHD patients, as reviewed by Saric et al. , points to the necessity of integrating genomic data with clinical history for better therapeutic targeting. They also suggested that investigating the interactions among ciliary genetics, CHD, and neurodevelopment could improve therapeutic management.

## Conclusion

In conclusion, this Research Topic advances our understanding of CHD by elucidating its genetic causes, improving diagnostics and therapeutics, and identifying gaps and challenges. These studies provide essential insights for greater diagnostic precision and new directions for enhancing CHD care and management. It also highlights multidisciplinary collaboration and leveraging of cutting-edge technologies will continue to drive innovations in CHD research, leading to better patient care and improved clinical outcomes.
